# Biallelic FBXW7 knockout induces AKAP8-mediated DNA damage in neighbouring wildtype cells

**DOI:** 10.1038/s41420-023-01494-y

**Published:** 2023-06-29

**Authors:** Dedrick Kok Hong Chan, Amit Mandal, Svenja Hester, Zhanru Yu, Geoff Stuart Higgins, Benedikt Mathias Kessler, Roman Fischer, Simon James Alexander Buczacki

**Affiliations:** 1 Nuffield Department of Surgical Sciences, University of Oxford, Oxford, UK; 2 NUS Centre for Cancer Research, Yong Loo Lin School of Medicine, Singapore, Singapore; 3 Nuffield Department of Medicine, Target Discovery Institute, University of Oxford, Oxford, UK; 4 Nuffield Department of Medicine, Chinese Academy for Medical Sciences Oxford Institute, University of Oxford, Oxford, UK; 5 Department of Oncology, University of Oxford, Oxford, UK

**Keywords:** Cancer genomics, Colorectal cancer

## Abstract

Colorectal cancer possesses marked intratumoral heterogeneity. While subclonal interactions between Vogelstein driver mutations have been extensively studied, less is known about competitive or cooperative effects between subclonal populations with other cancer driver mutations. *FBXW7* is a cancer driver mutation which is present in close to 17% of colorectal cancer cells. In this study, we generated isogenic *FBXW7* mutant cells using CRISPR-Cas9. We identified an upregulation of oxidative phosphorylation and DNA damage in *FBXW7* mutant cells, which surprisingly proliferated at a decreased rate compared to wildtype cells. To determine subclonal interactions, wildtype and mutant *FBXW7* cells were cocultured using a Transwell system. Wildtype cells cocultured with *FBXW7* mutant cells similarly developed DNA damage which was not observed when wildtype cells were co-cultured with other wildtype cells, suggesting that *FBXW7* mutant cells were inducing DNA damage in neighbouring wildtype cells. Using mass spectrometry, we identified AKAP8 as being secreted by *FBXW7* mutant cells into the coculture media. Furthermore, overexpression of AKAP8 in wildtype cells recapitulated the DNA damage phenotype observed during coculture, while co-culture of wildtype cells with double mutant *FBXW7*^*−/−*^*/AKAP8*^*−/−*^ cells abrogated the DNA damage phenotype. Here, we describe a hitherto unknown phenomenon of AKAP8-mediated DNA damage from *FBXW7* mutant to neighbouring wildtype cells. Our findings demonstrate the importance of elucidating the local effect of cancer driver mutations between subclonal populations.

## Introduction

Colorectal cancer (CRC) has been shown to possess marked intratumoral heterogeneity [1, 2], where subclonal populations harbouring different mutations coexist with each other. Recently, there has been increased interest in the clonal dynamics between these subclonal populations [3]. *Apc* mutant cells have been described to secrete the Wnt antagonist NOTUM into the tumour microenvironment, resulting in increased fitness of *Apc* mutant intestinal stem cells over wildtype counterparts [4, 5]. Given the dynamic interplay between subclonal populations, there is impetus to delineate the mechanisms surrounding such interactions. While there has been extensive research on the molecular interactions in Vogelstein driver mutations such as *APC* [4, 5], *KRAS* [6], *TP53* [7], and *SMAD4* [8], the elucidation of molecular mechanisms occurring in other cancer driver mutations has been less extensively studied. Multiregional genome and exome sequencing of both colon adenomas and CRC has revealed *FBXW7* to be a truncal and branch-driver mutation [1]. Based on The Cancer Genome Atlas (TCGA) data for CRC adenocarcinoma (COAD-READ), the incidence of *FBXW7* mutations in CRC was found to be 17.5% [9].

F-box and WD repeat domain containing 7 (FBXW7) is the substrate recognition component of the Skp1-Cdc53/Cullin–F-box–protein complex (SCF/β-TrCP) [10]. The SCF complex is the terminal interaction of a target protein in the ubiquitin-proteosome system (UPS) just prior to degradation. In this SCF complex, FBXW7 plays the important role of recognising proteins which have been phosphorylated at specific residues of a conserved CDC4 phosphodegron (CPD) motif. Following phosphorylation of target substrates via a glycogen synthase kinase 3 beta (GSK3β) process [11], FBXW7 recruits the target protein to the SCF complex where the target protein undergoes ubiquitination. This poly-ubiquitinated protein is then transported to the 26S proteasome where it undergoes proteolytic degradation [12].

FBXW7 has been shown to recognise at least 90 different substrates [13]. Many of these substrates are protooncogenes, and are key regulators of gene transcription and stem cell differentiation. Some of these targets include c-Myc, Cyclin E and Notch1. Mutations of *FBXW7* prevent the degradation and termination of pathways involving these proteins, leading to unabated cancer cell proliferation, and is the most commonly mutated protein in the SCF complex amongst human cancer cells [14]. In colorectal cancer (CRC), *FBXW7* is a driver mutation which has been associated with tumour initiation and progression [15]. CRC is also the second most common cancer to harbour *FBXW7* mutations. More recently, *FBXW7* mutations have also been found in normal colonic epithelium [16]. Given the central importance which *FBXW7* plays in CRC oncogenesis, we sought to uncover competitive or cooperative effects between subclones harbouring an *FBXW7* mutation and subclones wildtype for *FBXW7*.

In this study, we defined one mechanism of interaction between mutant *FBXW7* and wildtype subclones. We identified that *FBXW7* mutants upregulate metabolism via oxidative phosphorylation. DNA damage was upregulated in cells which were mutant for *FBXW7*, but strikingly, was also observed in neighbouring wild-type cells. We propose that A-kinase anchoring protein 8 (AKAP8), a member of the protein kinase A-anchoring protein family, which operates exclusively in the nucleus, is secreted in the microenvironment of *FBXW7* mutant cells, and could contribute to the increased DNA damage observed in *FBXW7* wildtype cells.

## Results

### *FBXW7*^*−/−*^ cells are at a proliferative disadvantage compared to wildtype cells

**Fig. 1:**
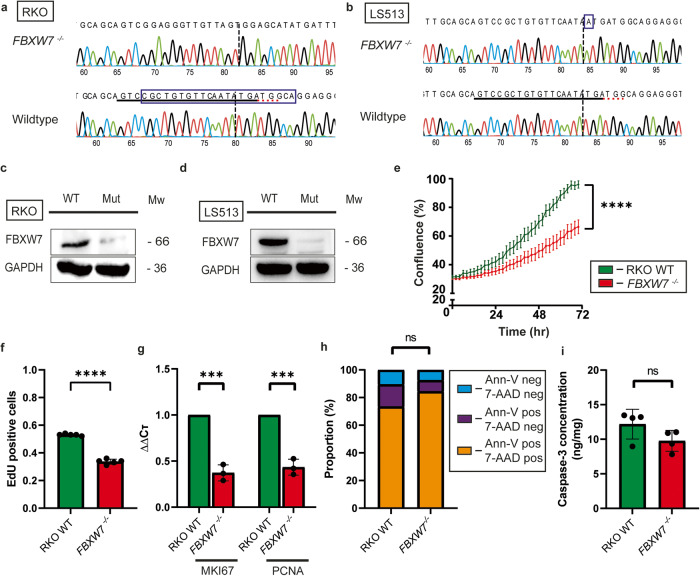
Characterisation of wildtype and *FBXW7*^*−/−*^ cells. **a** Sanger sequencing trace of RKO cell line showing an isogenic *FBXW7*^*−/−*^ clone with 22 nucleotide deletion (blue box) from wildtype. **b** Sanger sequencing trace of LS513 cell line showing an isogenic *FBXW7*^*−/−*^ clone with 1 nucleotide insertion (blue box) from wildtype. **c** Western blot validation showing loss of FBXW7 protein in RKO cells. **d** Western blot validation showing loss of FBXW7 protein in LS513 cells. **e** Live-cell proliferation analysis using IncuCyte imaging system showed increased proliferation of wildtype cells (green) relative to *FBXW7*^*−/−*^ cells (red). (*p* < 0.0001). **f** EdU incorporation assay showed increased proliferation occurring in wildtype cells relative to *FBXW7*^*−/−*^ cells (*p* < 0.0001). **g** RT-PCR showed increased MKI67 (*p* = 0.0002) and PCNA (*p* = 0.0003) in wildtype relative to *FBXW7*^*−/−*^ cells. **h** No statistically significant differences in both early and late apoptotic markers (blue: Annexin V and 7-AAD negative; violet: Annexin V positive, 7-AAD negative; orange: Annexin V and 7-AAD positive). **i** No difference in cleaved caspase 3 levels to suggest increased apoptosis accounting for the observed difference in cellular proliferation (*N* = 3). The values in this figure are mean ± sd, and statistical significance was measured by unpaired *t*-test. All experiments were performed with *N* = 3 biological replicates.

To evaluate the role of *FBXW7* subclones on neighbouring populations, isogenic *FBXW7*^−/−^ truncating mutations targeting the hotspot R505C on exon 10 were generated in two cell lines, RKO and LS513, using CRISPR-Cas9 and validated using Sanger sequencing (Fig. 1a and b) and western blot (Fig. 1c and d). RKO and LS513 were chosen to reflect the molecular diversity of CRC, LS513 being a microsatellite stable (MSS) cell line, while RKO exhibits microsatellite instability (MSI) [17]. Recently, p53 was found to be a substrate recognised by FBXW7 and degraded by the SCF complex [18–20]. Following ionising radiation or chemically mediated DNA damage, substrate recognition of p53 was found to be enhanced, resulting in increased degradation of p53, and ultimately cancer cell survival. Given the importance of the FBXW7–p53 relationship to cancer cell survival, both cell lines were also chosen as they were wildtype for *TP53*.

Surprisingly, we observed that *FBXW7*^*−/−*^ cells exhibited reduced proliferation compared to wildtype cells (Fig. 1e). Given FBXW7’s role in substrate recognition for the SCF complex, we had anticipated that knocking out *FBXW7* would result in upregulation of downstream mediators such as c-Myc, Cyclin E and Notch1 and lead to increased cellular proliferation. This observation was confirmed with a reduction in the EdU incorporation rate on flow cytometry (Fig. 1f and Supplementary Fig. 1a) and decreased relative expression of the proliferation markers *MKI67* and *PCNA* on reverse transcription-polymerase chain reaction (RT-PCR) (Fig. 1g) in *FBXW7*^*−/−*^ cells. On an extreme limiting dilution clonogenic assay, *FBXW7*^*−/−*^ cells were also observed to form fewer colonies compared with their wildtype counterparts (Supplementary Fig. 1b). We considered that the decreased proliferation observed could be accounted for by increased apoptosis. The proportion of cells positive for Annexin V and 7-amino-actinomycin (7-AAD) on flow cytometry was not found to be significantly different between wildtype and *FBXW7*^*−/−*^ cells (Fig. 1h). Enzyme-linked immunosorbent assay (ELISA) for cleaved caspase 3 (Fig. 1i) and cathepsin B (Supplementary Fig. 1c) was also not significantly different, as was a quantitative calpain activity assay (Supplementary Fig. 1d). Taken together, these findings suggest that *FBXW7*^*−/−*^ cells proliferated slower than wildtype cells, and this was not due to increased apoptosis.

### *FBXW7*^*−/−*^ demonstrates upregulation of oxidative phosphorylation

**Fig. 2:**
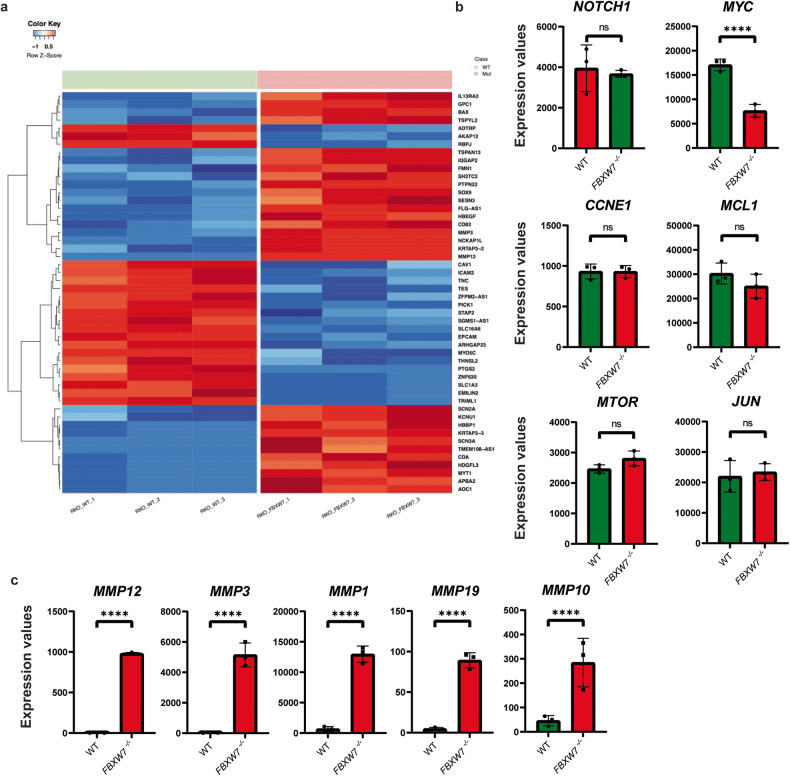
RNAseq results between wildtype and *FBXW7*^*−/−*^ cells. **a** Heatmap showing the top 50 differentially expressed genes between wildtype and *FBXW7*^*−/−*^ cells. **b** Transcript levels for genes which act as direct substrates for *FBXW7*. All differences were non-significant except for *MYC* (*p* = 0.0008) which was unexpectedly decreased in an *FBXW7* knockout, possibly due to negative feedback homeostatic regulation. **c** Transcript differences in metalloproteinases which were observed to be upregulated in *FBXW7*^*−/−*^ cells. All were significant with *p* < 0.0001. The values in this figure are mean ± sd, and statistical significance was measured by unpaired *t*-test. Biological triplicates, *N* = 3, were submitted for RNAseq.

To further molecularly characterise the effect which knocking out *FBXW7* has on a tumour cell, we performed bulk RNA sequencing (RNAseq) on wildtype and *FBXW7*^*−/−*^ RKO cells. Using a cut-off of log_2_FC > 1.0 or <−1.0 and *p* < 0.05, 362 genes were significantly upregulated, while 532 genes were downregulated (Fig. 2a). We observed that transcriptomic expression of the *FBXW7* gene was reduced in mutant cells compared with wildtype cells (Supplementary Fig. 1e). Among substrate targets of FBXW7, we noted that only *MYC* transcription was downregulated (Fig. 2b). This is likely a function of negative feedback homoeostatic regulation of c-Myc levels resulting from decreased ubiquitination of c-Myc by the SCF complex. C-Myc is known to be finely regulated at the transcriptional and translational levels [21, 22]. The half-life of *MYC* mRNA is ~15 min, and the mRNA is regulated by microRNAs (miRs) including miR-145, which inactivates the *MYC* mRNA in response to p53 [23]. Surprisingly, other substrate targets of FBXW7 were unchanged in *FBXW7*^*−/−*^ cells, which could account for the decreased proliferation of the cells relative to wildtype. Among other differentially expressed genes, we noticed that many matrix metalloproteinases (*MMP12*, *MMP3*, *MMP1*, *MMP19*, and *MMP10*) were significantly upregulated in *FBXW7*^*−/−*^ cells (Fig. 2c). MMPs have the ability to remodel the extracellular matrix (ECM), and their activity has been linked to a host of oncogenic processes including proteolytic degradation of the ECM, angiogenesis and eventual metastases [24]. Among metastatic colorectal cancers, the presence of *FBXW7* mutations has been associated with worse prognosis, greater risk of recurrence, and worse disease-free and overall survival [25–27]. However, no previous studies have linked mutations in *FBXW7* and changes in MMPs.

**Fig. 3:**
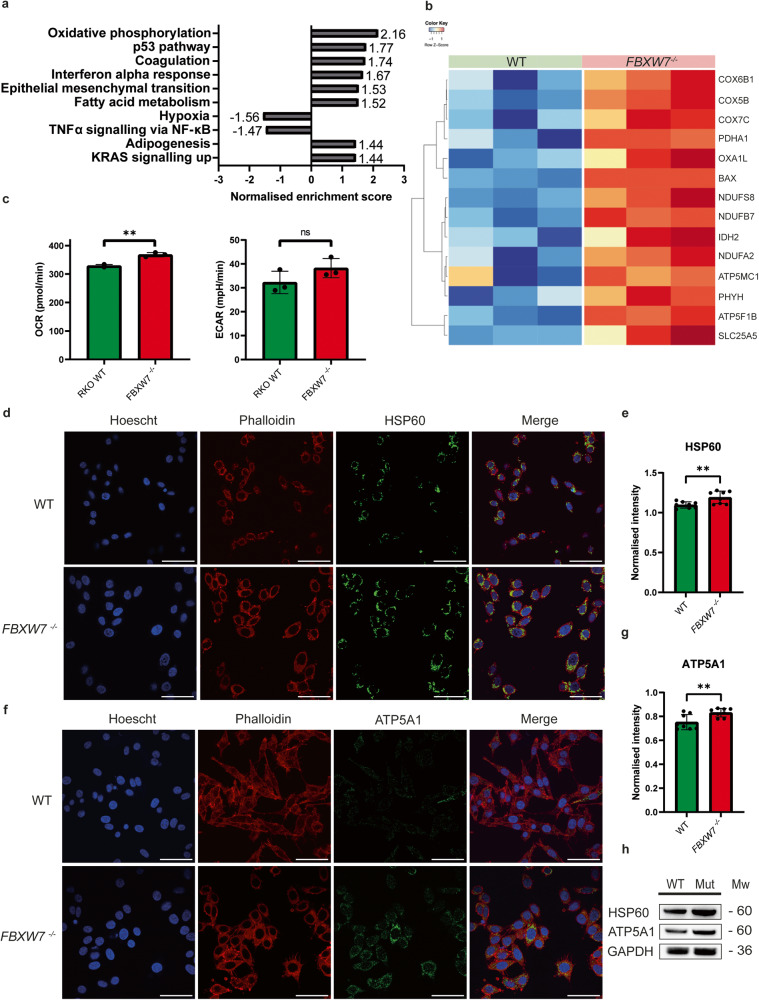
Oxidative phosphorylation is upregulated in *FBXW7*^*−/−*^ cells. **a** Analysis of Hallmark pathways using gene set enrichment analysis (GSEA) demonstrates that the oxidative phosphorylation pathway was the most upregulated gene set in *FBXW7*^*−/−*^ cells. This graph shows the top ten differentially expressed gene sets by normalised enrichment scores. **b** 86 genes within the oxidative phosphorylation gene set were differentially expressed. Among these 86, the 14 which showed log_2_FC > 0.7 or <0.7 have been represented in this heatmap. **c** Metabolic analysis of oxygen consumption rate was increased in *FBXW7*^*−/−*^ cells relative to wildtype cells (*p* = 0.0015). There was no significant difference in the extracellular acid production rate. **d** Immunofluorescence of wildtype and *FBXW7*^*−/−*^ cells with Hoechst 33342 nuclear stain (blue), phalloidin cytoskeleton stain (red) and HSP60 (green). Scale bar represents 50 µm. **e** Immunofluorescence of HSP60 was normalised against Hoechst and was increased in *FBXW7*^*−/−*^ cells (*p* = 0.0086). **f** Immunofluorescence of wildtype and *FBXW7*^*−/−*^ cells with Hoechst 33342 nuclear stain (blue), phalloidin cytoskeleton stain (red) and ATP5A1 (green). Scale bar represents 50 µm. **g** Immunofluorescence of ATP5A1 was normalised against Hoechst and was increased in *FBXW7*^*−/−*^ cells (*p* = 0.0085). **h** Western blot showing upregulation of HSP60 and ATP5A1 in *FBXW7*^*−/−*^ cells compared to wildtype cells. The values in this figure are mean ± sd, and statistical significance was measured by unpaired t-test. All experiments were performed with *N* = 3 biological replicates.

We proceeded to analyse the RNAseq data using gene set enrichment analysis (GSEA) for ‘Hallmark’ gene sets (50 gene sets), obtained from the molecular signatures database (MSigDB v4.0) [28], and found that the oxidative phosphorylation gene set showed the greatest magnitude of increase in terms of normalised enrichment score (NES) (Fig. 3a). Among the 200 genes included in the oxidative phosphorylation gene set, we observed an upregulation of 78 genes and a downregulation of 8 genes in *FBXW7*^*−/−*^ cells relative to wildtype. Among these 86 genes, 14 genes had a log_2_ fold change <0.7 or >0.7 (Fig. 3b). This finding suggested a decrease in the “Warburg effect”, in which aerobic glycolysis is upregulated in association with more aggressive cancers. Concordant with this association, we noted that the hypoxia hallmark gene set was downregulated in *FBXW7*^*−/−*^ cells (Fig. 3a). Taken together with our observation that *FBXW7*^*−/−*^ cells proliferated slower than wildtype cells, these findings suggest that *FBXW7* mutations may result in a reduction of the cell’s survival relative to its wildtype counterparts. Our study therefore not only validates earlier bioinformatic analyses based on the TCGA dataset which suggested that *FBXW7* mutations in a spectrum of various cancers cause metabolic reprogramming by upregulating oxidative phosphorylation, but we also demonstrate through RNAseq that this metabolic reprogramming is the major downstream effect of knocking out the *FBXW7* gene on the cell [29].

To validate our finding of increased oxidative phosphorylation, we studied the metabolic activity of cells in terms of the oxygen consumption rate (OCR) and extracellular acid production rate (ECAR), as surrogates for oxidative phosphorylation and glycolysis, respectively. We noted a significant increase in the OCR only (Fig. 3c). HSP60 is a chaperone protein which is required for the formation of the mitochondrial respiratory chain complex [30], while ATP5A is the catalytic subunit in complex V which generates adenosine triphosphate (ATP) from adenosine diphosphate (ADP). The upregulation in oxidative phosphorylation was therefore further validated with immunofluorescent staining (Fig. 3d–g) and western blot (Fig. 3h) for both proteins.

### *FBXW7*^*−/−*^ induce DNA damage in neighbouring wildtype cells

**Fig. 4:**
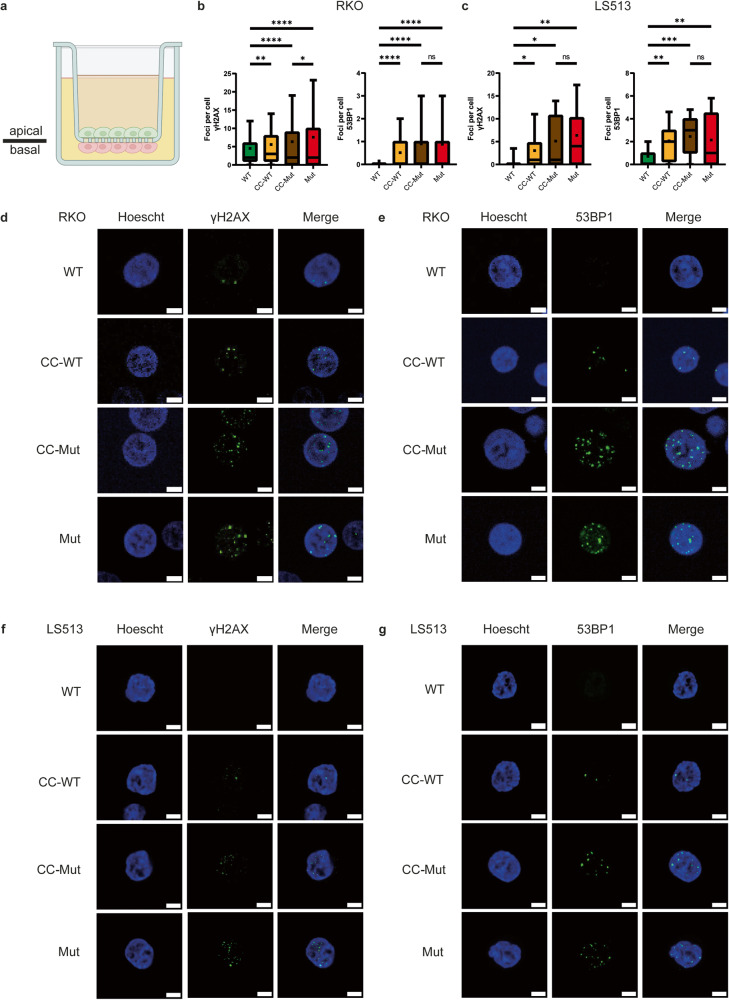
DNA damage was increased in wildtype cells which were co-cultured with *FBXW7*^*−/−*^ cells. **a** Schematic showing how cells were cultured on the Transwell insert. Cells are cultured on both sides of the insert and are therefore in close proximity with one another without cells being able to migrate across the membrane. **b** In RKO cells, γH2AX foci per nuclei were significantly increased between wildtype and all other groups. Notably, γH2AX foci were increased between WT and CC-WT cells. (WT vs. CC-WT, *p* = 0.0032; WT vs. CC-Mut, *p* < 0.0001; WT vs. CC-Mut^*-*^, *p* < 0.0001^*;*^ CC-Mut vs. Mut, *p* = 0.0154.) This same effect was observed when staining for 53BP1 foci. (WT vs. CC-WT, *p* < 0.0001; WT vs. CC-Mut, *p* < 0.0001; WT vs. Mut, *p* < 0.0001; CC-Mut vs. Mut, ns.) **c** In LS513 cells, γH2AX foci per nuclei were significantly increased between wildtype and all other groups. As in RKO cells, γH2AX was increased between WT and CC-WT cells (WT vs. CC-WT, *p* = 0.0404; WT vs. CC-Mut, *p* = 0.0189; WT vs. Mut, *p* = 0.0029; CC-Mut vs. Mut, ns). A similar effect was observed when staining for 53BP1 foci (WT vs. CC-WT, *p* = 0.0022; WT vs. CC-Mut, *p* = 0.0004; WT vs. Mut, *p* = 0.0052; CC-Mut vs Mut, ns). Immunofluorescent images of nuclei with γH2AX foci in RKO cells (**d**) and LS513 cells (**f**). Immunofluorescent images of nuclei with 53BP1 foci in RKO cells (**e**) and LS513 cells (**g**). All scale bars represent 5 µm. Boxplots are presented as 10–90 percentiles with the median value represented by a line across the box, and an asterisk to denote the mean. Statistical significance was measured by unpaired *t*-test. All experiments were performed with *N* = 3 biological replicates.

In the process of generating ATP from oxygen, oxidative phosphorylation is known to result in the production of reactive oxygen species (ROS), which can in turn result in DNA damage [31, 32]. Indeed, we observed that *FBXW7*^*−/−*^ cells harboured significantly increased DNA damage compared to wildtype cells on immunofluorescent staining for DNA damage markers using anti-γH2AX and anti-53BP1 in both RKO (Fig. 4b, d, e) and LS513 (Fig. 4c, f, g) cells.

Given the association between *FBXW7* mutations in CRC and worse overall prognosis [27, 33], we were surprised to observe decreased proliferation in our *FBXW7*^*−/−*^ cells. This led us to consider the effect of *FBXW7*^*−/−*^ cells on its neighbouring cancer cells. CRC carries significant intratumoral heterogeneity where subclones with different driver mutations compete constantly with one another [3]. To determine the effect of the *FBXW7*^*−/−*^ cells on neighbouring wildtype cells, we cultured mutant and wildtype cells on either surface on the insert of a Transwell (Corning, New York, USA) plate (Fig. 4a). We selected an insert pore size which allowed media to freely permeate between both compartments of the Transwell, without allowing for cell migration. To ensure all cells were treated uniformly, wildtype cells were cocultured with wild-type cells and are denoted WT cells. *FBXW7*^*−/−*^ were also cocultured with *FBXW7*^*−/−*^ cells and denoted Mut cells. Wildtype cells cocultured with *FBXW7*^*−/−*^ cells were harvested separately and denoted CC-WT and CC-Mut cells respectively. Coculture experiments were performed for both RKO and LS513 cell lines. Intriguingly, coculturing showed an upregulation of DNA damage in CC-WT cells compared to WT cells. There was no difference in the burden of DNA damage between CC-Mut cells and Mut cells. These findings were validated with a comet assay which showed increased tail length as well as the tail moment in CC-WT cells compared with WT cells (Supplementary Fig. 2a–c).

Increased DNA damage may be attributed to the effect of CRISPR-Cas9, given that this approach to gene editing introduces a double-strand break in the DNA at the target site. To ensure the effects observed were not a function of CRISPR-Cas9 gene editing, we targeted the *AAVS1* safe harbour locus and performed single-cell cloning to obtain a clonal population (Supplementary Fig. 3a). Measurement of immunofluorescence for anti-γH2AX and anti-53BP1 between wildtype and *AAVS1* mutants did not show any difference, suggesting that the effect observed between CC-WT and CC-Mut cells was due to the introduction of the *FBXW7* knockout (Supplementary Fig. 3b–e). These findings suggest that *FBXW7*^*−/−*^ cells induce DNA damage in neighbouring wildtype cells with no observable reverse effect noted.

### AKAP8 from *FBXW7*^*−/−*^ mediates DNA damage on wildtype cells

**Fig. 5:**
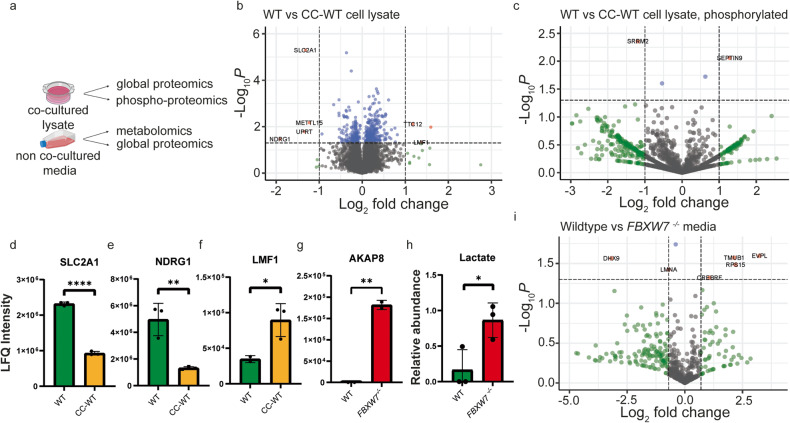
Proteomic analysis of wildtype and CC-WT cells. **a** Schematic showing a breakdown of sample types submitted for proteomic or metabolic analyses. **b** Volcano plot showing global proteomic differences between wildtype and CC-WT cells. Highlighted proteins had log_2_FC>1 or <-1 and *p* < 0.05. **c** Volcano plot showing phospho-proteomic differences between wildtype and CC-WT cells. Highlighted proteins had log_2_FC>1 or <-1 and *p* < 0.05. Individual LFQ intensity differences for **d** SLC2A1 (*p* < 0.0001), **e** NDRG1 (*p* = 0.0343), and **f** LMF1 (*p* = 0.0476). **g** AKAP8 was found in the *FBXW7*^*−/−*^ cell media but not in the wildtype media. The LFQ intensity difference is represented here (*p* = 0.0018). **h** In the metabolic analysis of media from wildtype and *FBXW7*^*−/−*^ cells, only lactate was found to be significantly increased in media from *FBXW7*^*−/−*^ cells. **i** Volcano plot showing proteomic differences between the media from wildtype and *FBXW7*^*−/−*^ cells for proteins which were present in both media fractions. Highlighted proteins had log_2_FC>0.7 or <-0.7 and *p* < 0.05. The values in this figure are mean ± sd, and statistical significance was measured by unpaired *t*-test. Proteomic and metabolic analyses were performed with *N* = 3 biological replicates.

Mass spectrometry was performed on cocultured cells to characterise the effect which co-culturing *FBXW7*^*−/−*^ and wildtype cells had. As many of the proteins involved in DNA damage and repair require phosphorylation, mass spectrometry was used to analyse the global proteome as well as the phospho-proteome (Fig. 5a).

When comparing between Mut and WT cells and applying a cut-off of log_2_FC > 2 or < −2 and *p* < 0.05, SERPINB9, HDAC2 and LUM were upregulated in Mut cells while ANXA1, MRS2, CMTR2, LMAN1, BIN3, RUNX1, TBP and MCFD2 were downregulated (Supplementary Fig. 4a). In colorectal cancer, ANXA1 (Annexin A1), has been shown to be associated with a worse prognosis and an increased immune cell infiltrate (Supplementary Fig. 4b) [34]. MCFD2 and LMAN1 form a protein complex which is responsible for shuttling cargo between the endoplasmic reticulum and the Golgi apparatus for transport out of the cell. Deficiencies in either protein have been shown to result in severe disease, including α1-antitrypsin deficiency [35] and factor V and factor VIII deficiency [36]. Combined downregulation of MCFD2 and LMAN1 (Supplementary Fig. 4c and d) suggests severely dysfunctional protein transport out of the cell in *FBXW7*^*−/−*^ cells and could be a novel effect of *FBXW7* knockout. Conversely, SERPINB9 and HDAC2 were observed to be upregulated in *FBXW7*^*−/−*^ cells and both have been described as good prognostic markers for CRC (Supplementary Fig. 4e and f) [37, 38]. Among proteins which were present in both Mut and WT cells, and applying a cut-off of log_2_FC > 1 or <−1 and *p* < 0.05, 7 proteins were found to be significantly downregulated in Mut compared with WT cells (Supplementary Fig. 5); none were significantly upregulated. Among proteins which were exclusively expressed in either WT or Mut cells, we observed 51 proteins found in cell lysates of WT cells only, and 25 found in cell lysates of Mut cells only.

When comparing CC-WT and WT cells and applying a cut-off of log_2_FC > 1 or <−1 and *p* < 0.05, STXBP2, TTC2, GEMIN2, LMF1, ADAMTSL1 and RBL2 were upregulated in CC-WT cells while SLC2A1, NDRG1, METTL15, UPRT and P4HA2 were downregulated (Fig. 5b, d–f). *SLC2A1* encodes for the glucose transporter 1 (GLUT1) protein which is an important mediator of glycolysis. GLUT1 functions by facilitating the diffusion of glucose molecules across cell membranes, and its expression closely mirrors glycolytic activity within cells [39]. Downregulation of GLUT1 in CC-WT cells suggests metabolic reprogramming away from glycolysis and towards oxidative phosphorylation which could account for the increase in DNA damage observed in CC-WT cells (Fig. 5d), as in *FBXW7*^*−/−*^ cells. We also observed the downregulation of NDRG1 and upregulation of LMF1 in CC-WT cells (Fig. 5e and f), which have both been found to be independently associated with a poorer prognosis in CRC [40, 41] suggesting that wildtype cells neighbouring *FBXW7*^*−/−*^ cells have a worse prognosis compared with wildtype cells by its own. Among phospho-proteins which were isolated in both CC-WT and WT cells, and applying a cut-off of log_2_FC > 1 or <−1 and *p* < 0.05, SRRM2 was downregulated in CC-WT cells, while SEPTIN9 was upregulated (Fig. 5c).

Given that increased DNA damage was observed in our Transwell coculture system, we hypothesised that the causative agent must be secreted by *FBXW7*^*−/−*^ cells and present in the coculture media. Furthermore, the causative agent of interest would have to be upregulated in media from *FBXW7*^*−/−*^ cells. We, therefore, harvested media from both wildtype and *FBXW7*^*−/−*^ cells and subjected this to metabolic and proteomic analysis. Among proteins which were found in media from both wildtype and *FBXW7*^*−/−*^ cells, applying a cut-off of log_2_FC > 0.7 or <−0.7 and *p* < 0.05, we identified that EVPL, TMUB1, RPS15 and CREBRF were upregulated in the media of *FBXW7*^*−/−*^ cells (Fig. 5i). In addition, AKAP8 was found exclusively in *FBXW7*^*−/−*^ cells and not in wildtype cells (Fig. 5g). Metabolic analysis of media from both cell types did not yield significant differences, except for lactate (Fig. 5h). This likely reflected the increased metabolic demand in *FBXW7*^*−/−*^ cells.

**Fig. 6:**
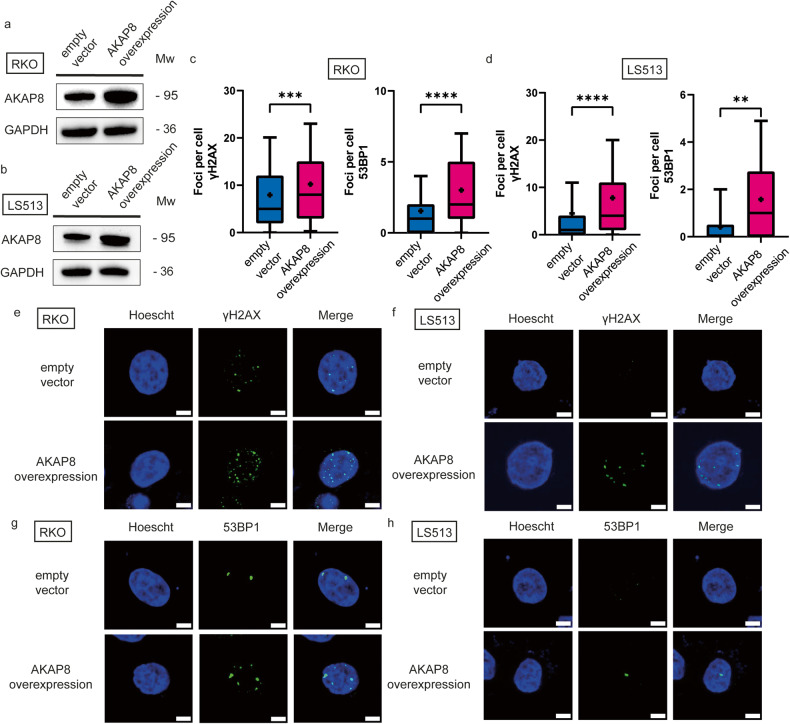
AKAP8 overexpression induces DNA damage in wildtype cells. AKAP8 was overexpressed in wildtype cells and observed for DNA damage. Western blot demonstrating upregulation of AKAP8 in RKO cells (**a**) and LS513 cells (**b**). **c** In RKO cells, γH2AX foci were upregulated between cells transfected with an empty vector and cells with the AKAP8 overexpression vector (*p* = 0.0008). 53BP1 was also upregulated in cells with the AKAP8 overexpression vector (*p* < 0.0001). **d** Similar results were observed in LS513 cells for γH2AX (*p* < 0.0001) and 53BP1 (*p* = 0.0026). Immunofluorescent images of nuclei with γH2AX foci in RKO cells (**e**) and LS513 cells (**f**). Immunofluorescent images of nuclei with 53BP1 foci in RKO cells (**g**) and LS513 cells (**h**). All scale bars represent 5 µm. Boxplots are presented as 10–90 percentiles with the median value represented by a line across the box, and an asterisk to denote the mean. Statistical significance was measured by unpaired *t*-test. All experiments were performed with *N* = 3 biological replicates.

Given that AKAP8 was the only protein to have been exclusively secreted into the media of *FBXW7*^*−/−*^ cells, we sought to investigate whether AKAP8 was the putative agent causing increased DNA damage in wildtype cells. This was performed via two modalities. Firstly, we overexpressed AKAP8 with a pcDNA3.1(+)-AKAP95-FRB plasmid. The pcDNA3.1 with an empty vector was used as a control. Western blot in both RKO and LS513 cells demonstrated upregulation of AKAP8 in the cell population with the overexpression plasmid (Fig. 6a and b). Immunofluorescence of γH2AX and 53BP1 was observed to be upregulated in both RKO (Fig. 6c, e, g) and LS513 (Fig. 6d, f, h).

**Fig. 7:**
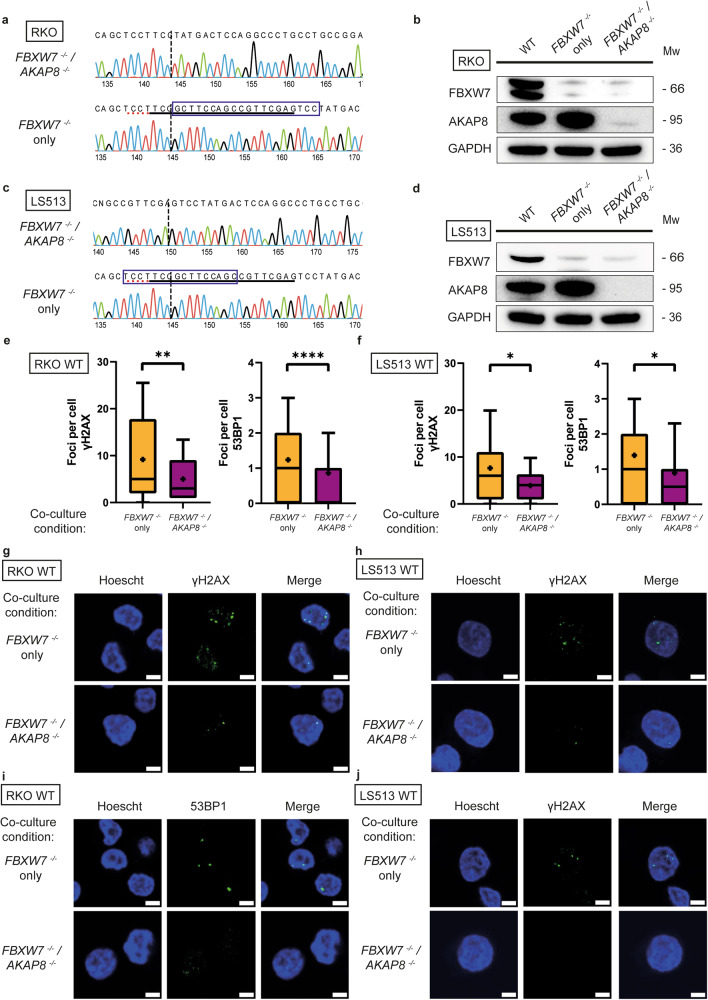
Loss of AKAP8 protein abrogates the DNA damage effect seen in neighbouring cells. **a** Sanger sequencing trace of RKO cell line showing an isogenic *AKAP8*^*−/−*^ clone with 20 nucleotide deletion (blue box) from wildtype. **b** Western blot validation showing loss of FBXW7 or AKAP8 protein in RKO cells. **c** Sanger sequencing trace of LS513 cell line showing an isogenic *AKAP8*^*-/-*^ clone with 16 nucleotide deletion (blue box) from wildtype. **d** Western blot validation showing loss of FBXW7 or AKAP8 protein in LS513 cells. RKO wildtype cells were co-cultured with either single mutant *FBXW7*^*−/−*^ cells or double mutant *FBXW7*^*−/−*^*/AKAP8*^*−/−*^ cells. In RKO cells, γH2AX (*p* = 0.0011; Fig. 7e, g) and 53BP1 (*p* < 0.0001; Fig. 7e, i) foci were decreased in double mutant cells compared with single mutant cells. Similarly, LS513 wildtype cells were co-cultured with either single mutant *FBXW7*^*−/−*^ cells or double mutant *FBXW7*^*−/−*^*/AKAP8*^*−/−*^ cells. In LS513 cells, γH2AX (*p* = 0.0285; Fig. 7f, h) and 53BP1 (*p* = 0.0479; Fig. 7f, j) foci were decreased in double mutant cells compared with single mutant cells. Boxplots are presented as 10–90 percentiles with the median value represented by a line across the box, and an asterisk to denote the mean. Statistical significance was measured by unpaired *t*-test. All experiments were performed with *N* = 3 biological replicates.

In a second approach, we tested whether AKAP8 was a crucial agent in the secretome of *FBXW7*^*−/−*^ mutant cells resulting in DNA damage of neighbouring wildtype cells. This was achieved by introducing a mutation in the *AKAP8* gene of *FBXW7*^*−/−*^ mutant cells. Double mutant isogenic *FBXW7*^*−/−*^/*AKAP8*^*−/−*^ cells were therefore generated in the two cell lines using CRISPR-Cas9 and the knockout mutation was validated using Sanger sequencing and western blot (RKO: Fig. 7a and b; LS513: Fig. 7c and d). Wildtype cells were co-cultured either with single mutant *FBXW7*^*−/−*^ or double mutant *FBXW7*^*−/−*^/*AKAP8*^*−/−*^ cells. Immunofluorescence of γH2AX and 53BP1 was observed to be decreased in wildtype cells co-cultured with double mutant *FBXW7*^*−/−*^/*AKAP8*^*−/−*^ cells compared with single mutant *FBXW7*^*−/−*^ cells in both RKO (Fig. 7e, g, i) and LS513 (Fig. 7f, h, j) cells, suggesting a crucial role for AKAP8 in inducing DNA damage in neighbouring wildtype cells.

### Effects of *FBXW7* mutation are recapitulated in TCGA-COAD data

**Fig. 8:**
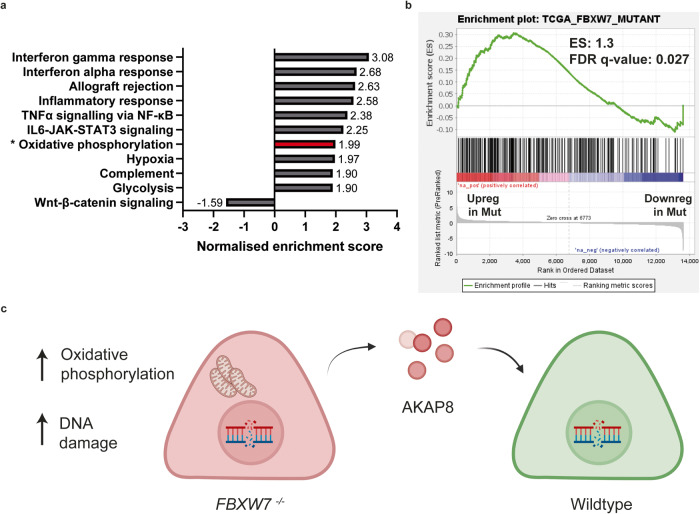
Our *FBXW7* cell line model correlates with TCGA-COAD-derived patient samples. **a** Analysis of Hallmark pathways using GSEA demonstrates that the oxidative phosphorylation pathway was also upregulated in TCGA-COAD-derived *FBXW7* mutant cancers. The top 10 differentially expressed gene sets are represented here, with the addition of the most differentially expressed gene set which had a negative correlation in *FBXW7* mutants. For Oxidative phosphorylation (highlighted with asterisk, and bar in red), the NES was 1.99, with an FDR *q*-value < 0.0001. **b** Enrichment plot depicting a significant correlation between the gene set associated with TCGA-COAD-derived *FBXW7* mutant patient samples, and our own RKO cell line-derived bulk RNAseq data. **c** Schematic showing the interaction between *FBXW7*^*−/−*^ and wildtype cells. *FBXW7*^*−/−*^ cells demonstrate increased oxidative phosphorylation metabolism as well as an increased accumulation of DNA damage. *FBXW7*^*−/−*^ cells secrete AKAP8 into its microenvironment, and this is taken up by cells which are wildtype for *FBXW7*. This results in increased DNA damage in wildtype cells.

Finally, we analysed TCGA data to determine if the results from our study were recapitulated in tumour data. Comparing transcriptomic differences between patients with wildtype and mutant *FBXW7*, and using a *q*-value cut-off of <0.05, we observed 302 genes with increased expression in mutant *FBXW7*, and 394 genes with increased expression in wildtype *FBXW7* cells. GSEA for ‘Hallmark’ gene sets (50 gene sets), obtained from the molecular signatures database (MSigDB v4.0) showed that the oxidative phosphorylation gene set was indeed significantly upregulated in mutant cells, with an NES of 1.99, and a false discovery rate (FDR) *q*-value of < 0.0001 (Fig. 8a).

The aforementioned 302 genes which were significantly upregulated in *FBXW7* cells were grouped as a “TCGA_FBXW7_mutant” gene set. GSEA from our bulk RNAseq analysis comparing RKO wildtype and *FBXW7*^*−/−*^ cells were ranked against this “TCGA_FBXW7_mutant” gene set (Fig. 8b). We observed a good correlation between our *FBXW7* model and TCGA-derived patient data of *FBXW7* mutant cells, suggesting that results from our experiments using our *FBXW7* cell line models may be extrapolated to real-world patient data.

## Discussion

AKAP8, also known as AKAP95, is one of over 50 different types of A-kinase anchoring proteins whose main function is to mediate cAMP-dependent protein kinase A (PKA) activity. PKA activity is governed by two regulatory subunits, while its effect is mediated by two catalytic subunits. The binding of AKAPs to PKAs confines the activity of PKA to a narrow subset of potential substrates [42]. AKAP8 is primarily localised to the nucleus. The association of AKAP8 to PKA, as well as PKA-mediated cAMP signalling, is responsible for maintaining chromatin in a condensed state during mitosis [43]. Independent of PKA, AKAP8 has also been shown to regulate DNA replication [44] and to promote tumorigenesis and increased proliferation by binding to cyclin D and cyclin E during the G1/S phase [45]. More recently, RNAseq performed on MDA-MB-231 cells in which AKAP8 was knocked down showed on gene ontology analysis that DNA repair genes were most significantly upregulated [46], suggesting that AKAP8 is involved in the transcription of DNA repair genes and that overexpression in our case, could lead to downregulation of DNA repair genes and therefore the accumulation of DNA damage. Our research, therefore, showed that wildtype cells neighbouring *FBXW7*^*−/−*^ cells accumulate DNA damage via AKAP8 (Fig. 8c).

Notably, we were unable to relate the finding of AKAP8 secretion by *FBXW7*^*−/−*^ cells back to the upregulation in oxidative phosphorylation observed in *FBXW7*^*−/−*^ cells, we used an unbiased approach to uncover cellular interactions at the microenvironmental level. In spite of this, fundamentally, our work unravels a novel mechanism of interaction between subclones in cancer. We have demonstrated that the phenotype of a mutant may be inherited by virtue of being in the microenvironment of the mutant subpopulation. In this case, *FBXW7*^*−/−*^ cells which demonstrate increased DNA damage are able to transmit this damage to neighbouring cells which do not possess the *FBXW7* mutation. This finding has significant implications for subclonal interactions. For example, a dominant subclone could potentially imbue neighbouring subclones with phenotypes which would otherwise not have been expected based on the mutational profile of those cells. Our findings suggest that further unpicking mechanisms which exist at the subclonal level could provide major insights into tumour development and therapeutic vulnerabilities.

## Materials and methods

### Cell culture

RKO and LS513 cells were purchased from ATCC(USA). Cells were cultured in DMEM/F12 (Gibco) supplemented with 10% FBS (Gibco) and 100 U/ml of penicillin–streptomycin (Gibco). Cells were subcultured when 80% confluent by incubating with TrypLe (Gibco). Mycoplasma testing was carried out regularly using PlasmoTest Mycoplasma Detection Kit (InvivoGen). Cells were cultured in a humidified 5% CO_2_ chamber at 37 °C.

### Generation of clonal CRISPR-Cas9 gene knockouts

sgRNAs were purchased from Synthego for both the *FBXW7* gene and to target the control AAVS1 region (Table 1). We used an electroporation approach which has been described in detail previously [47]. Briefly, cells were dissociated into single-cell suspension using TrypLe (Gibco). A ribonucleoprotein (RNP) complex was generated by mixing 25 µM of Cas9 enzyme (Sigma-Aldrich), and 100 µM of the sgRNA to generate a molar ratio of 1:1 after mixing. Using a Neon Transfection System (ThermoFisher), cells were electroporated with the RNP complex at voltage 1300 V, width 20 ms and 2 pulses. Cells were then incubated for one week. After one week, transfection efficacy was checked by Sanger sequencing (Genewiz), using an amplicon generated with the forward and reverse primers during polymerase chain reaction (PCR).

**Table 1:** sgRNA sequences and primers used for validation of gene knockout.

sgRNA sequences for CRISPR-Cas9	
Gene target	sgRNA sequence
*FBXW7*	GUCCGCUGUGUUCAAUAUGA
AAVS1 (*PPP1R12C*)	GGGGCCACUAGGGACAGGAU
*AKAP8*	CUCGAACGGCUGGAAGCGGA

Sequencing primers to validate gene knockout		
Gene target	Forward primer	Reverse primer
*FBXW7*	TCTGCAGAGTTGTTAGCGGT	TGGACTGTACTGGATCAGCAA
AAVS1 (*PPP1R12C*)	GGTCCGAGAGCTCAGCTAGT	GGCTCCATCGTAAGCAAACC
*AKAP8*	AGTGTTCCAGGGAGGGTGTT	GGGTCTCGGCATTCACTGTA

To obtain a clonal culture, cells were serial diluted to one cell/20 µl and 20 µl of the cell suspension was transferred to each well of a 96-well plate. Cells were expanded and each colony was Sanger sequenced again to pick colonies which showed isogenic knockout of the target gene or region of interest.

### Protein lysates and western blot

Proteins were harvested using RIPA (ThermoFisher) with the addition of protease and phosphatase inhibitor cocktails (Sigma-Aldrich). Quantification of protein concentration was performed using BCA assay (ThermoFisher).

For the western blot, 30 µg of protein was used for each lane. Proteins were separated using a NuPage 4–12% Bis-Tris gel (Invitrogen) and transferred onto a 0.45 µm nitrocellulose membrane. The membrane was blocked with 5% low-fat milk for 1 h before overnight incubation with primary antibodies at 4 °C. Rabbit anti-human FBXW7 antibody (1:2500, BS-8394R, Bioss, USA), rabbit anti-human ATP5A antibody (1:2500, ab176569, Abcam, USA), rabbit anti-human HSP60 antibody (1:2500, ab46798, Abcam, USA), rabbit anti-human AKAP95 antibody (1:2500, ab134923, Abcam, USA for Fig. 6; 1:2500, ab72196, Abcam, USA for Fig. 7), rabbit anti-human GAPDH antibody (1:2500, ab9485, Abcam, USA) was used. The membrane was then incubated with goat anti-rabbit antibody (1:5000, ab6721, Abcam, USA) at room temperature for 1 h. The membranes were then imaged with a ChemiDoc XRS+ system (Bio-Rad).

### Quantitative reverse transcription-PCR

Total RNA was extracted using RNeasy Mini Kit (Qiagen) as per the manufacturer’s protocol. cDNA was synthesised using SuperScript IV VILO Master Mix (Invitrogen). Real-time qPCR was then performed using TaqMan Fast Advanced Master Mix (Applied Biosystems) and TaqMan probes (MKI67, Hs04260396_g1; PCNA, Hs00427214_g1) on a StepOnePlus Real-Time PCR System (Applied Biosystems).

### Live-cell microscopy

Cells were seeded at density 5 × 10^4^/ml in a 24-well plate and observed at ×10 magnification every 2 h for 72 h in an IncuCyte (Sartorius) imaging system.

### Flow cytometry

To measure for proliferating cells, a Click-iT™ EdU Alexa Fluor™ 647 Flow Cytometry Assay Kit (Invitrogen) was used. Briefly, EdU was added to the cells at 10 μM for 2 h. Click-iT reaction was performed after cells were fixed and permeabilised. Live/dead staining was performed with propidium iodide prior to flow cytometry.

To measure cell apoptosis, an Annexin V Apoptosis Kit with 7-AAD, FITC (Stemcell Technologies) was used. Briefly, 1 × 10^6^ cells were stained with Annexin V Binding Buffer before Annexin V-FITC and 7-AAD were added to the cells for 15 min at room temperature while protected from light. Cells were then imaged via flow cytometry.

All flow cytometry was performed on a CytoFLEX (Beckman Coulter) flow cytometer and analysed with the CytExpert 2.4 software (Beckman Coulter).

### Extreme limiting dilution colony forming assay

Cells were seeded at the following densities 5, 25, 50, 100, 200, 300, 400 and 500 cells/ml and incubated for 72 h. Colonies were then counted manually and analysed using the webtool interface http://bioinf.wehi.edu.au/software/elda/ [48].

### Metabolic assay

Metabolic assay was performed using the Agilent Seahorse Real-Time ATP Rate Assay kit and in accordance with the manufacturer’s instructions. This assay was performed on the Seahorse Agilent XFe96 Analyzer. Briefly, equal quantities of wildtype and *FBXW7*^*−/−*^ cells were seeded on Seahorse XF microplate. Cell densities ranging from 0.5 × 10^4^ cells/ well to 4 × 10^4^ cells/well in increments of 0.5 × 10^4^ cells were seeded the day before to ensure good adhesion of cells to the culture plates. A sensor cartridge was hydrated at 37 ^o^C in a non-CO_2_ incubator the day before. On the day of the experiment, Seahorse XF DMEM, pH 7.4 was supplemented with 10 mM of XF glucose, 1 mM of XF pyruvate, and 2 mM of XF glutamine and warmed to 37 ^o^C. Previous media from the Seahorse XG microplate was then removed and freshly prepared Seahorse XF DMEM with supplements was added. Cells were incubated in a non-CO_2_ incubator for 60 min at 37 ^o^C before Seahorse XF DMEM media with supplements was refreshed. To determine the OCR and ECAR, 1.5 µM of oligomycin and 0.5 µM of Rotenone/Antimycin A were added to the cells 20 and 40 min after the initiation of the analyzer, respectively. Measurements of metabolism were made every 6.5 min. Mean values of the OCR and ECAR for each cell density of wildtype and *FBXW7*^*−/−*^ cells were calculated.

### Apoptosis assays

For quantification of cleaved Caspase-3, the Caspase 3 (Cleaved) Human ELISA Kit (Invitrogen) was used. Briefly, 5 × 10^6^ cells were harvested and resuspended in cell lysis buffer (Invitrogen) supplemented with protease inhibitor cocktail (Sigma-Aldrich) and PMSF protease inhibitor (Thermo Scientific). After centrifugation, the supernatant was collected and diluted 1:10, before being transferred to the antibody-coated plate. After the addition of a detector antibody, the anti-rabbit IgG HRP solution was then added with incubation for 30 min. Stabilised chromogen was then added and the stop solution was transferred to the wells after 30 min. Absorbance at 450 nm was measured on a plate reader immediately. All steps were performed at room temperature.

For the quantification of Cathepsin B, the Human Cathepsin B ELISA Kit (Abcam) was used. Briefly, 5 × 10^6^ cells were harvested and resuspended in cell lysis buffer (Invitrogen) supplemented with protease inhibitor cocktail (Sigma-Aldrich) and PMSF protease inhibitor (Thermo Scientific). After centrifugation, the supernatant was collected and diluted 1:10, before being transferred to the antibody-coated plate. The biotinylated anti-human Cathepsin B antibody was then added to each well and incubated for 90 min. After washing, the avidin–biotin–peroxidase complex working solution was then added to wells for 40 min. After washing, TMB colour-developing agent was then added for 30 min, and the reaction was arrested with the TMB stop solution. Absorbance at 450 nm was measured on a plate reader immediately. All steps were performed at room temperature.

For the measurement of calpain activity, the Calpain Activity Assay Kit (Abcam) was used. Briefly, 1 × 10^6^ cells were harvested and resuspended in extraction buffer on ice for 20 min. Protein concentration was then measured and 100 µg of protein was used for each assay reaction. Calpain substrate was subsequently added to each well and incubated for 60 min at 37 °C. Fluorescence following excitation at 400 nm was then measured on a plate reader immediately.

All measurements were recorded on a POLARstar Omega microplate reader (BMG Labtech).

### RNA sequencing and analyses

Total RNA was extracted using RNeasy Mini Kit (Qiagen) as per the manufacturer’s protocol. RNA quantity and quality were measured using a NanoDrop One Microvolume UV–Vis Spectrophotometer (ThermoFisher Scientific) and Agilent 2100 Bioanalyzer, respectively. Sequencing was performed by Oxford Genomics Centre on an Illumina NextSeq500 instrument using the standard paired-end protocol with a read length of 150 bp. Fastq reads were processed to clip low-quality leading and trailing edges and remove any adaptor content using Cutadapt (v.3.5). Quality-checked fastq reads were then aligned to the human genome (GRCh38) and gene annotation (Ensembl release 105) using STAR aligner (v.2.7.3a) two-pass mode to generate gene-level quantification. Raw counts were then processed in R (v.4.1.2) for all statistical testing and plotting purposes. Normalisation and differential expression were performed using limma (v.3.50.0), and pathway level significance testing was performed using fgsea (v.1.20.0) on MSigDb (Molecular Signature Database, v.7.5.1) hallmark pathways. Heatmaps have been plotted using gplots (v.3.1.1).

### Transwell cell culture

24 mm diameter Transwell inserts, made of polyester and with a pore size of 0.4 µm were used. These were placed in six-well plates. 3 × 10^6^ cells were cultured on each side of the insert. 1 ml of media was placed in the insert, while 2 ml of media was placed in the receiver.

### Immunofluorescence and imaging

Cells were cultured on an 8-well µ-Slide (ibidi) at a density of 3 × 10^4^ cells in each well. After fixation and permeabilization, cells were blocked with 2% BSA in PBS for one hour at room temperature. Cells were washed with PBS before the primary antibody was added and incubated for 2 h at room temperature. Cells were then washed with TBS before secondary antibody, nuclear, and cytokeratin stains were added and incubated for 45 min at room temperature. Imaging was performed directly on the slides with the Andor Dragonfly High-Speed Confocal Microscope Systems (Oxford Instruments). A list of antibodies and concentrations used in this project is included in Table 2.

**Table 2:** Antibodies and concentrations of antibodies used for immunofluorescence.

Target	Concentration	Brand	Cat#
HSP60	1:1000	Abcam	Ab46798
ATP5A1	1:1000	Invitrogen	459240
γH2AX	1:2500	Abcam	Ab81299
53BP1	1:2500	Abcam	Ab172580
Phalloidin 647	1:1000	Abcam	Ab176759
Hoescht 33342	1 µg/ml	Thermo Scientific	62249
Anti-rabbit	1:1000	Abcam	Ab150077
Anti-mouse	1:1000	Abcam	Ab150113

Quantification of fluorescent intensity for HSP60 and ATP5A1 was done using ImageJ. The pipeline for speckle counting on CellProfiler4 was used to perform foci counting of γH2AX and 53BP1 [49].

### Single-cell gel electrophoresis assay

Single-cell gel electrophoresis was performed using the CometAssay(biotechne) as per the manufacturer’s instructions. Briefly, 50 µl of cells at a concentration of 1 × 10^5^/ml were placed onto a CometSlide(biotechne) and allowed to spread evenly across the sample area. Slides were incubated at 4 °C in the dark for 10 min. Slides were then immersed in lysis solution for 60 min at 4 °C, before being transferred to Alkaline unwinding solution for 20 min at room temperature in the dark. Slides were placed on an electrophoresis slide tray and submerged in 4 °C Alkaline electrophoresis solution. The power supply was set at 21 V, and run for 30 min. After electrophoresis was run, slides were immersed gently in distilled water twice, and then in 70% ethanol for 5 min. Samples were dried at 37 °C for 15 min. 100 µl of 1X SYBR Gold was placed over the dried agarose and allowed to stain for 30 min before being rinsed in distilled water. Fluorescence microscopy was performed immediately. Quantification of comet assay parameters was performed using the pipeline for comet assays on CellProfiler4 [49].

### Proteomics analysis

Proteins were harvested using RIPA (ThermoFisher) with the addition of protease and phosphatase inhibitor cocktails (Sigma-Aldrich). Quantification of protein concentration was performed using BCA assay (ThermoFisher). The media fraction was obtained from media in which cells had been growing for 4 days. Media was aspirated and transferred into a centrifuge tube, and subjected to centrifugation at 1200 rpm. The supernatant was then transferred into a clean tube.

Filter-aided sample preparation (FASP) was performed. Briefly, 20 µg of lysate and 2.5 ml of media were loaded onto a Vivacon 500 (Sartorius) filter and spun at 14, 300 rcf for 10 min. Samples were denatured with 8 M urea for 30 min at room temperature followed by reduction with 10 mM Tris(2-carboxyethyl)phosphine(TCEP) and 50 mM Iodacetamide for 30 min at room temperature. Two cycles of washes with 200 µl 50 mM TEAB were applied to reduce the 8 M urea to <1 M prior to trypsin addition. 2 µg of trypsin in 200 µl 50 mM TEAB was added and incubated overnight at 37 °C. The next day, peptides were eluted from the Vivacon 500 filter with 200 µl 0.1% trifluoroacetic acid (TFA)/water and 200 µl 50% acetonitrile (ACN)/water. Eluate was then dried in a SpeedVac Vacuum Concentrator (ThermoFisher).

For mass spectrometry, peptides were resuspended in 5% formic acid and 5% DMSO and then trapped on an Acclaim™ PepMap™ 100 C18 HPLC Columns (PepMapC18; 300 µm × 5 mm, 5 µm particle size, ThermoFisher) using solvent A (0.1% formic acid in water) at a pressure of 60 bar and separated on an Ultimate 3000 UHPLC system (ThermoFisher) coupled to a QExactive mass spectrometer (ThermoFisher). The peptides were separated on an Easy Spray PepMap RSLC column (75 µm i.d. × 2 µm × 50 mm, 100 Å, ThermoFisher) and then electro-sprayed directly into an QExactive mass spectrometer (Thermo Fischer Scientific) through an EASY-Spray nano-electrospray ion source (ThermoFisher) using a linear gradient (length: 60 min, 5–35% solvent B (0.1% formic acid in acetonitrile and 5% dimethyl sulfoxide), flow rate: 250 nL/min). The raw data was acquired in the mass spectrometer in a data-independent mode (DIA). Full scan MS spectra were acquired in the Orbitrap (inclusion list with scan range 495–995*m*/*z*, 20*m*/*z* increments, with an overlap of ±2 Da, resolution 35000, AGC target 3e6, maximum injection time 55 ms). After the MS scans peaks were selected for HCD fragmentation at 28% of normalised collision energy(nce)/stepped nce. HCD spectra were also acquired in the Orbitrap (resolution 17500, AGC target 1e6, isolation window 20*m*/*z*).

For data analysis, the search engine used was DIA-NN 1.8 in a library-free search mode. For protein identification, peaks were searched against the database UPR_*Homo sapiens*_9606_UP000005640 with the following parameters—deep learning-based spectra, RTs and IMs prediction, the maximum number of protease (trypsin) missed cleavages set to 1, post-translational modifications: Carbamidomethyl (C), Oxidation (M), false discovery rate (FDR) set to 1%, and match between runs was used.

### Metabolomics analysis

Metabolomic analysis was performed by GCxGC–MS essentially as described [50].

In brief, the extraction of metabolites was carried out at temperature ambient in two steps from 100 μL of media by using 1 mL of tert-butyl methyl ether (MTBE) and 800 μL methanol respectively. Myristic acid-14,14,14-D3 was used as an internal standard. Homogenisation was performed using a bead beater (Precellys 24, Bertin Technologies). The supernatant of MTBE and methanol were combined and dried in vacuo (Speed Vac Centrifugation, Thermofisher).

The dried samples were resuspended in a solution of methoxyamine hydrochloride in pyridine (50 μl, 20 μg/μL) and shaken (1200 rpm) for 90 min at 30 °C. 70 μL N-Methyl-N-trimethylsilyltrifluoroacetamide (MSTFA) with 1% chlorotrimethylsilane (TMCS) and 30 μL pyridine were added to the samples, followed by incubation for one hour at 60 °C at a shaking speed of 1200 rpm. The samples were cooled down to temperature ambient and injected directly for GCxGC–MS analysis.

The derivatized samples were immediately analysed using a GCxGC–MS system comprising a gas chromatograph coupled to a quadrupole mass spectrometer (Shimadzu GCMS QP2010 Ultra) and a Shimadzu AOC-20i/s autosampler. The first-dimensional separation was carried out on an SHM5MS capillary column (30 m × 0.25 mm i.d. × 0.25 μm film thickness, Shimadzu) while the second-dimensional separation was on a BPX-50 capillary column (5 m × 0.15 mm i.d. × 0.15 μm film thickness, SGE). Helium gas was used as a carrier gas. The modulation period was set as 4 s. The samples were injected at 280 °C. The oven temperature was programmed from 60 to 320 °C at 10 °C/min unless stated otherwise and held at 320 °C for 8 min. The interface temperature to the mass spectrometer was set at 300 °C and the ion source was heated at 230 °C. The MS was operated at scan speeds of 20,000 amu covering a range of *m*/*z* 45–600. Electron Ionisation spectra were recorded at 70 eV.

Raw GC×GC MS data were processed using GCMSsolution software (v2.72/4.20 Shimadzu), and Chromsquare software (v2.1.6, Shimadzu) in combination with the NIST 11, NIST 11/s, OA_TMS, FA_ME and YUTDI in-house libraries were used for data analysis. The annotation of metabolites was carried out by comparing them to external standards (IM spectra and retention times adjusted to the internal standard myristic acid-14,14,14-d3) and by spectrum matching-based searches with the above databases for those metabolites without external standards. The similarity score threshold was set to 80 (out of 100), and the confidence of identification was further validated by manual inspection of matches between experimentally observed and reference EI spectra. Transient transfection of plasmid for AKAP8 overexpression.

pcDNA3.1(+)-AKAP95-FRB was a gift from Jin Zhang (Addgene plasmid # 138254; RRID:Addgene_138254) [51]. pcDNA3.1(+) empty vector was a gift from David Kerr. Plasmids were extracted using PureYield™ Plasmid Miniprep System (Promega). Transient transfection of cells was achieved with Lipofectamine 3000 (Invitrogen).

### Statistical analyses

Statistical analysis was performed using GraphPad Prism 9. All data are presented as mean ± standard deviation (sd). *p*-values < 0.05 were considered significant. Significance was further defined as follows: *p < 0.05,**p < 0.01, ***p < 0.001, ****p < 0.0001. For comparisons between two groups with parametric data, a two-tailed unpaired Students’ *t*-test was used for each comparison. In the figure legends, N denotes the number of biological replicates. Boxplots have been presented as 10–90 percentiles with the median value represented by a line across the box, and an asterisk to denote the mean. All depicted data points are biological replicates taken from distinct samples.

## Supplementary information

Supplementary Figure 1

Supplementary Figure 2

Supplementary Figure 3

Supplementary Figure 4

Supplementary Figure 5

Supplemental figure legends

## Data Availability

All datasets will be made available to readers promptly on request.
