# Supplementary figures and images for: Biallelic FBXW7 knockout induces AKAP8-mediated DNA damage in neighbouring wildtype cells
Source: Cell Death Discov. 2023 Jun 29;9:200. doi: 10.1038/s41420-023-01494-y (PMC10310709; doi:10.1038/s41420-023-01494-y)

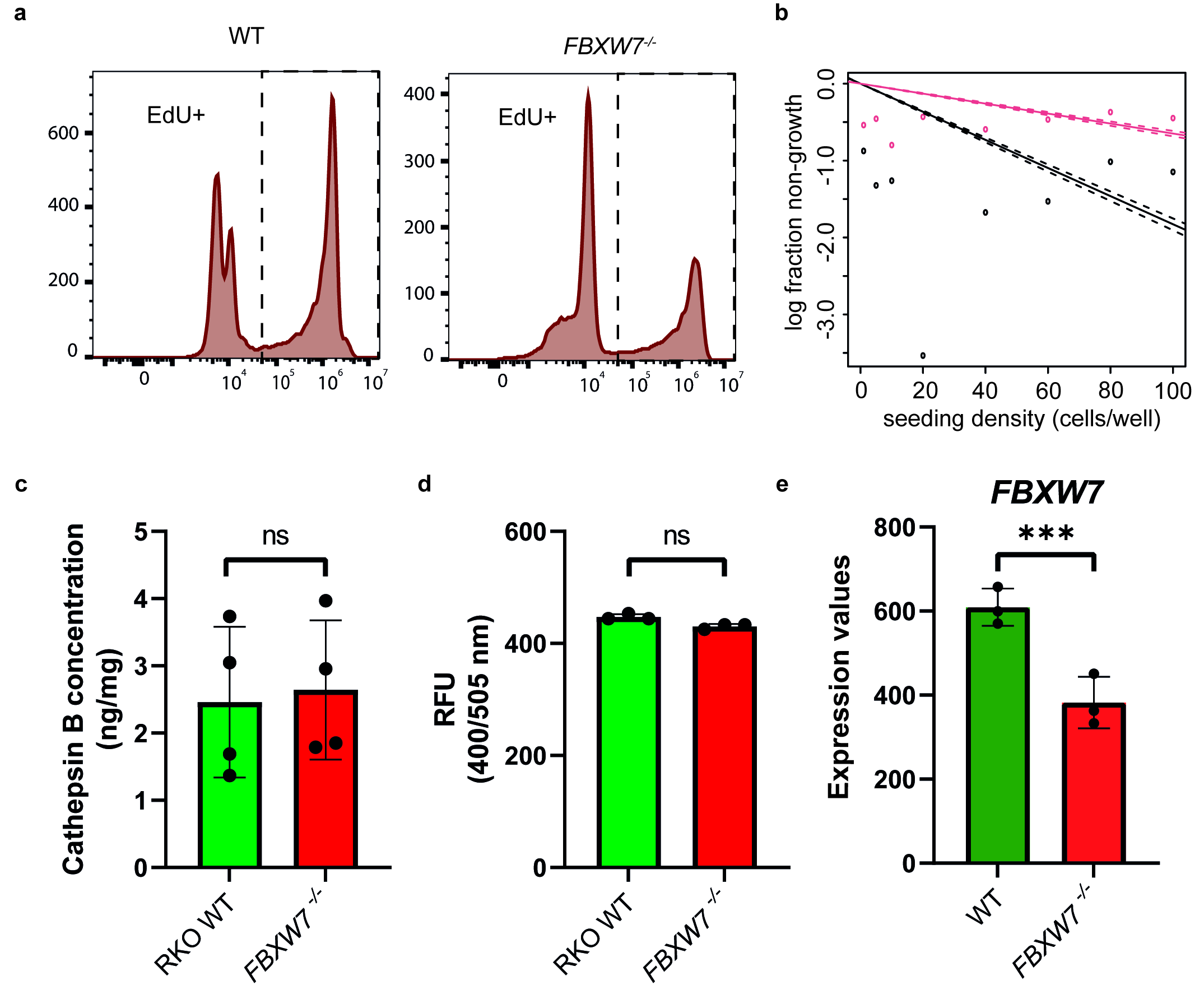

Supplement: Supplementary file 1 — Supplementary Figure 1 [file 41420_2023_1494_MOESM1_ESM.tif]

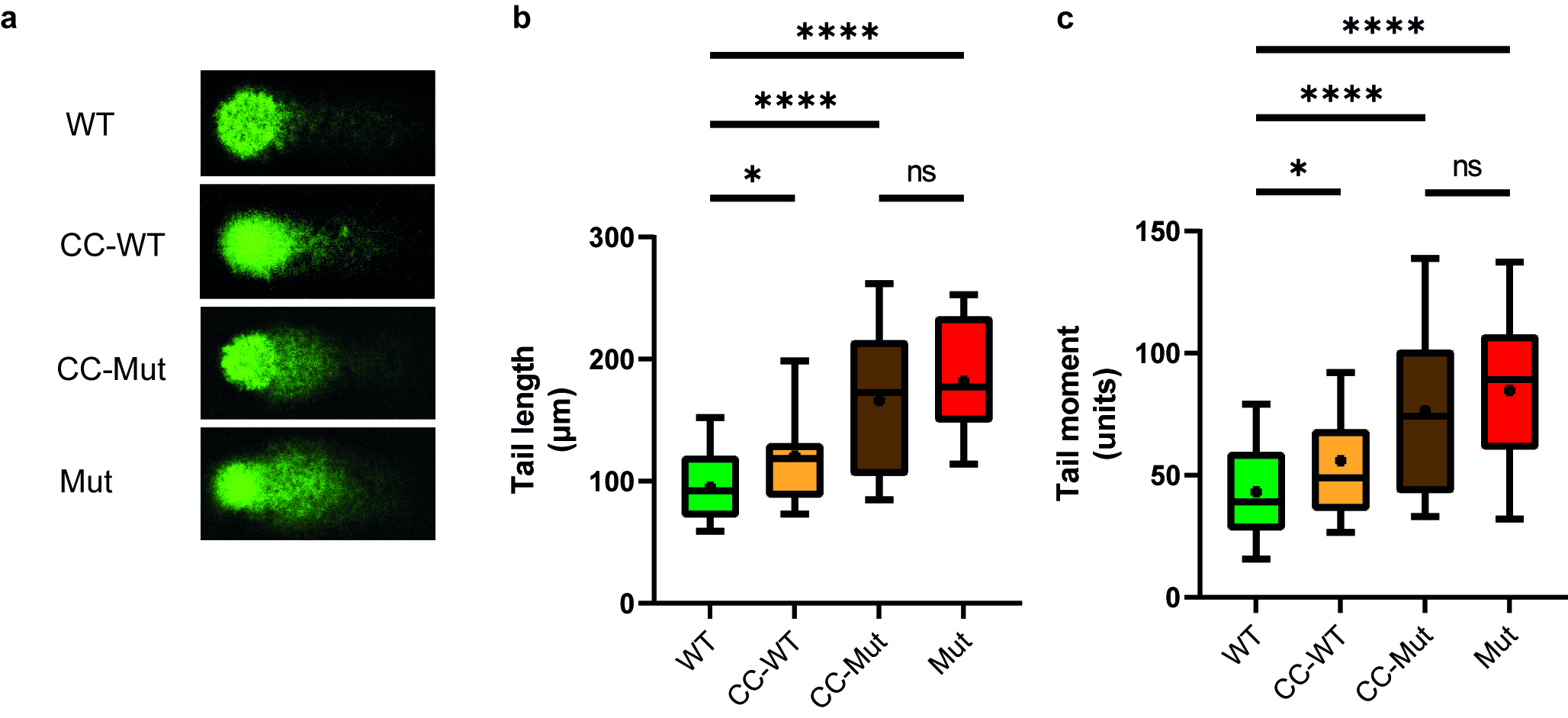

Supplement: Supplementary file 2 — Supplementary Figure 2 [file 41420_2023_1494_MOESM2_ESM.tif]

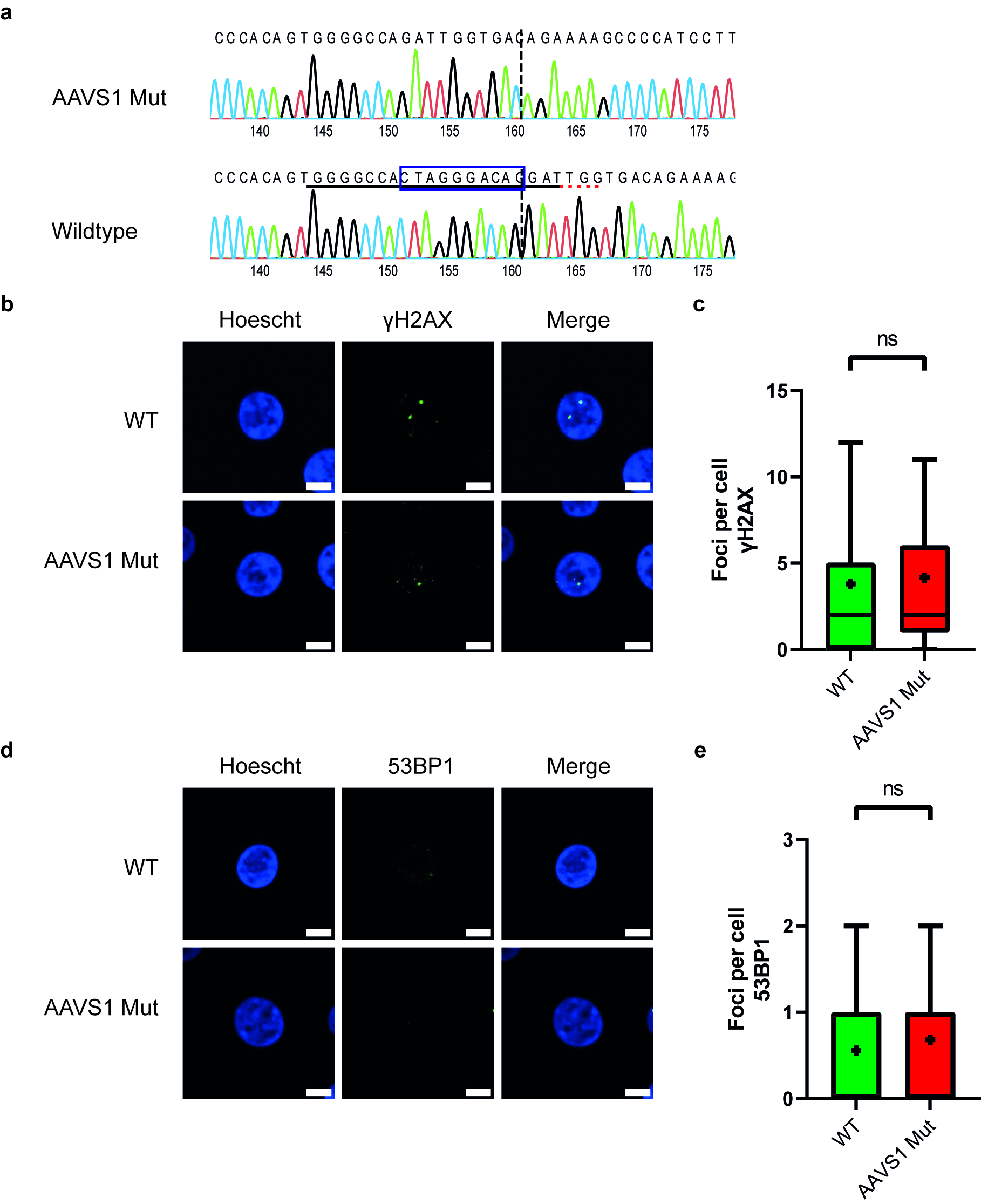

Supplement: Supplementary file 3 — Supplementary Figure 3 [file 41420_2023_1494_MOESM3_ESM.tif]

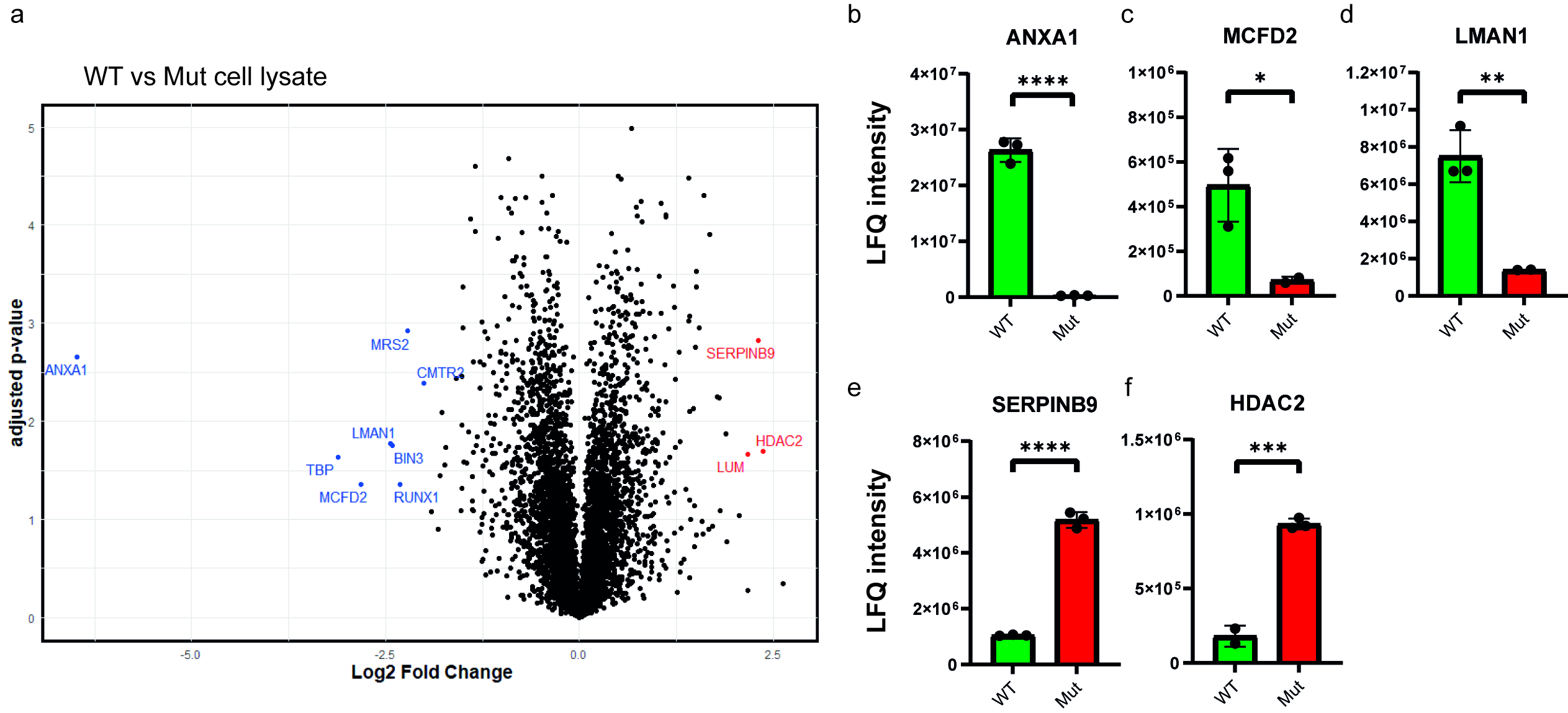

Supplement: Supplementary file 4 — Supplementary Figure 4 [file 41420_2023_1494_MOESM4_ESM.tif]

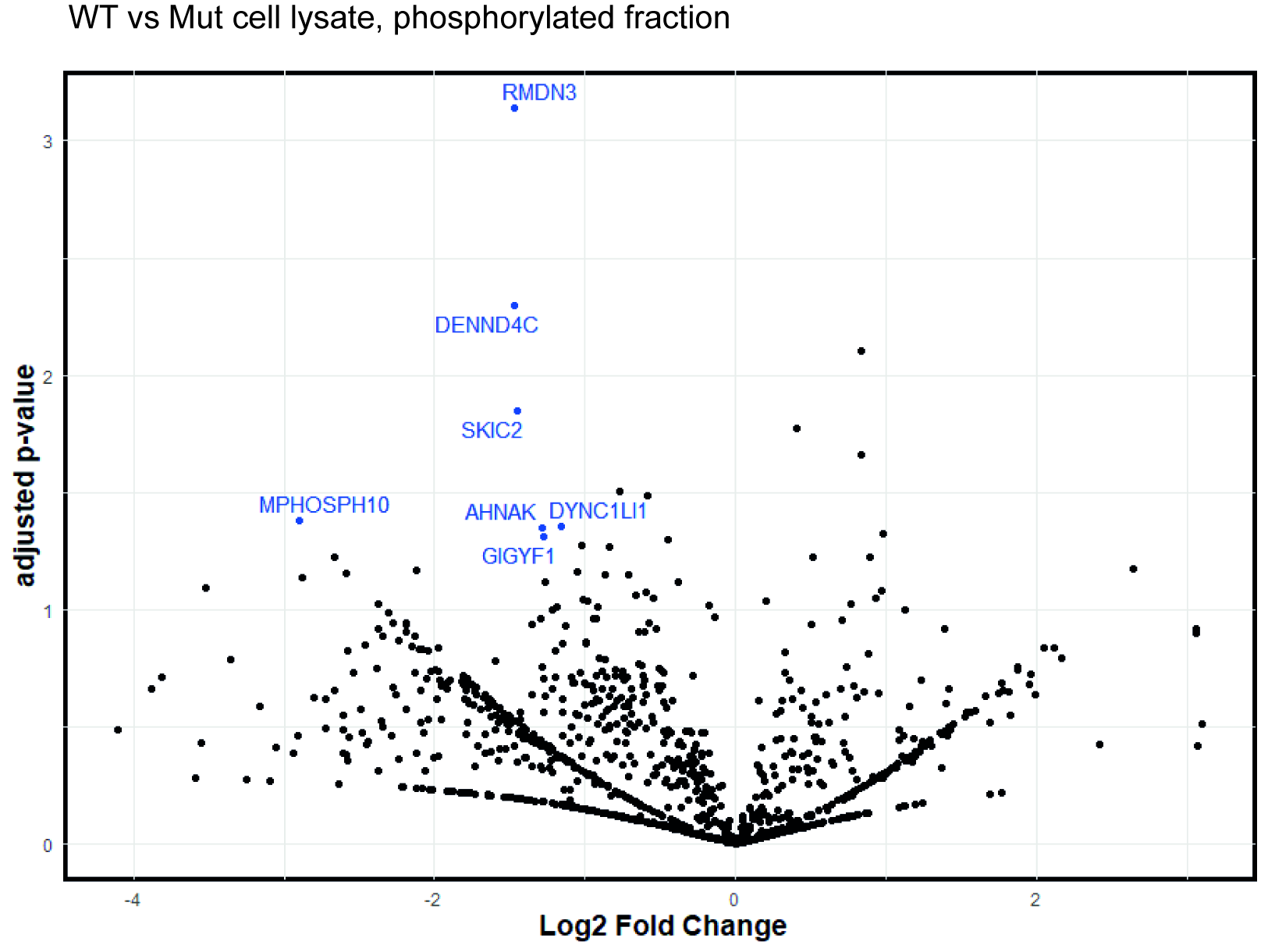

Supplement: Supplementary file 5 — Supplementary Figure 5 [file 41420_2023_1494_MOESM5_ESM.tif]
