# Supplementary material for: Biallelic FBXW7 knockout induces AKAP8-mediated DNA damage in neighbouring wildtype cells
Source: Cell Death Discov. 2023 Jun 29;9:200. doi: 10.1038/s41420-023-01494-y (PMC10310709; doi:10.1038/s41420-023-01494-y)
Supplement: Supplementary file 6 — Supplemental figure legends [file 41420_2023_1494_MOESM6_ESM.docx]

Supplementary figure legends:

Supplementary figure 1: Characterisation of wildtype and *FBXW7^-/-^* cells. a. Histogram showing gating thresholds to determine EdU positivity. b. Results from clonogenic assay showing the increased ability of wildtype cells (black) to form clones relative to *FBXW7^-/-^* cells (red) (*p*< 0.0001). c. No difference in Cathepsin B between wildtype and *FBXW7^-/-^* cells. d. No difference in Calpain activity between wildtype and *FBXW7^-/-^* cells. e. Transcript level for *FBXW7* was significantly decreased in *FBXW7^-/-^* cells compared to wildtype on bulk RNAseq (*p*= 0.0065). The values in this figure are mean ± sd, and statistical significance was measured by unpaired t-test. All experiments were performed with N = 3 biological replicates.

Supplementary figure 2: Comet assay showed DNA damage was increased in wildtype cells co-cultured with *FBXW7^-/-^* cells. a. Single-cell gel electrophoresis images for WT, CC-WT, CC-Mut and Mut cells. b. Tail length was significantly increased between wildtype and all other groups. Notably, tail length was increased between WT and CC-WT cells (WT vs CC-WT, *p*= 0.0185; WT vs CC-Mut, *p*< 0.0001; WT vs Mut, *p*< 0.0001; CC-Mut vs Mut, ns). c. A similar relationship was observed for tail moment (WT vs CC-WT, *p*= 0.0367; WT vs CC-Mut, *p*< 0.0001; WT vs Mut, *p*< 0.0001; CC-Mut vs Mut, ns). Boxplots are presented as 10 – 90 percentiles with the median value represented by a line across the box, and an asterisk to denote the mean. Statistical significance was measured by unpaired t-test. All experiments were performed with N = 3 biological replicates.

Supplementary figure 3: AAVS1 knockouts did not show increased DNA damage. a. Sanger sequencing trace showing an isogenic AAVS1 knockout with 10 nucleotide deletion (blue box) from wildtype. b. Immunofluorescent images of nuclei with γH2AX foci showing no difference in the number of foci per nuclei (c). d. Immunofluorescent images of nuclei with 53BP1 foci showing no difference in the number of foci per nuclei (e). All scale bars represent 5µm. Boxplots are presented as 10 – 90 percentiles with the median value represented by a line across the box, and an asterisk to denote the mean. Statistical significance was measured by unpaired t-test. All experiments were performed with N = 3 biological replicates.

Supplementary figure 4: Proteomic analysis of WT and Mut cells. a. Volcano plot showing proteomic differences between WT and Mut cells. Highlighted proteins had log_2_FC > or < 2 and p< 0.05. Individual LFQ intensity differences for (b) ANXA1 (*p*< 0.0001), (c) MCFD2 (*p*= 0.0396), (d) LMAN1 (*p*= 0.0098), (e) SERPINB9 (*p*< 0.0001), and (f) HDAC2 (*p*= 0.0005) are represented here. The values in this figure are mean ± sd, and statistical significance was measured by unpaired t-test. Proteomic analyses were performed with N = 3 biological replicates.

Supplementary figure 5: Phospho-proteomic analysis of WT and Mut cells. Volcano plot showing proteomic differences between WT and Mut cells. Highlighted proteins had log_2_FC > or < 1 and p< 0.05. Proteomic analyses were performed with N = 3 biological replicates.
